# Tigers of Sundarbans in India: Is the Population a Separate Conservation Unit?

**DOI:** 10.1371/journal.pone.0118846

**Published:** 2015-04-28

**Authors:** Sujeet Kumar Singh, Sudhanshu Mishra, Jouni Aspi, Laura Kvist, Parag Nigam, Puneet Pandey, Reeta Sharma, Surendra Prakash Goyal

**Affiliations:** 1 Department of Biology, University of Oulu, Oulu, Finland; 2 Department of Animal Ecology and Conservation Biology, Wildlife Institute of India, Dehradun, India; 3 Population and Conservation Genetics, Instituto Gulbenkian de Ciência, Rua da Quinta, 6, P-2780–156, Oeiras, Portugal; Estonian Biocentre, ESTONIA

## Abstract

The Sundarbans tiger inhabits a unique mangrove habitat and are morphologically distinct from the recognized tiger subspecies in terms of skull morphometrics and body size. Thus, there is an urgent need to assess their ecological and genetic distinctiveness and determine if Sundarbans tigers should be defined and managed as separate conservation unit. We utilized nine microsatellites and 3 kb from four mitochondrial DNA (mtDNA) genes to estimate genetic variability, population structure, demographic parameters and visualize historic and contemporary connectivity among tiger populations from Sundarbans and mainland India. We also evaluated the traits that determine exchangeability or adaptive differences among tiger populations. Data from both markers suggest that Sundarbans tiger is not a separate tiger subspecies and should be regarded as Bengal tiger (*P*. *t*. *tigris*) subspecies. Maximum likelihood phylogenetic analyses of the mtDNA data revealed reciprocal monophyly. Genetic differentiation was found stronger for mtDNA than nuclear DNA. Microsatellite markers indicated low genetic variation in Sundarbans tigers (*He*= 0.58) as compared to other mainland populations, such as northern and Peninsular (*He*between 0.67- 0.70). Molecular data supports migration between mainland and Sundarbans populations until very recent times. We attribute this reduction in gene flow to accelerated fragmentation and habitat alteration in the landscape over the past few centuries. Demographic analyses suggest that Sundarbans tigers have diverged recently from peninsular tiger population within last 2000 years. Sundarbans tigers are the most divergent group of Bengal tigers, and ecologically non-exchangeable with other tiger populations, and thus should be managed as a separate “evolutionarily significant unit” (ESU) following the adaptive evolutionary conservation (AEC) concept.

## Introduction

Tiger (*Panthera tigris*) conservation remains an enormous challenge at both national and global scales and its long-term persistence depends on effective conservation and management strategies. Five extant tiger subspecies have been recognized on the basis of geographic distribution and morphological characteristics [1]. However, molecular genetics approach applied by Luo et al. [2] elucidated the presence of six phylogeographic groups or subspecies of the tiger.

On the basis of tiger distribution and the potential for connectivity, India is divided into six tiger landscape complexes [3]. One of them “Sundarbans landscape complex” represents the last stronghold of Bengal tigers adapted to living in mangrove forests [3, 4, 5]. Although never examined in detail, Sundarbans tigers are considered as distinct from other subspecies and are traditionally assigned to Bengal tigers (*P*. *t*. *tigris*) [6]. The forest in Sundarbans is traversed by many tidal channels forming several small to large forest islands and is shared by India and Bangladesh [3]. By combining the results of camera traps and radio telemetry investigations, it has been estimated that the total number of tigers in the Indian part of Sundarbans is around 70 (CI: 64–90) [5]. Few studies have been performed to understand various aspects of tiger ecology in Sundarbans [7, 8]. However, there is no information about the genetic makeup and diversity of the Sundarbans tigers, which are threatened by habitat destruction, prey depletion, direct tiger loss (due to human-tiger conflict), and climate change [4, 9]. In order to investigate if Sundarbans tigers have characteristics that distinguish them from other mainland tiger populations, Barlow et al. [10] compared five tiger skulls from the Bangladesh Sundarbans with 175 skulls representing nine tiger subspecies (also including three that are already extinct). Surprisingly, they found that the skulls of Sundarbans tigers were smaller and significantly different from all other subspecies. They also found that the body weight of Sundarbans tigress varies from 75 to 80 kg, which is actually half of the body weight of the tigress in the mainland. Thus, morphological distinctiveness of Sundarbans tigers raised questions regarding their conservation status. Based on their results, the authors recommended that Sundarbans tigers should be managed separately and evaluated further to determine if they form an ESU.

Identification of population units within species is a crucial step for guiding management authorities and policy makers in management practices [11]. Delineation of these units have been a controversial issue over the last two decades and there is no general agreement on the criteria that define the two most commonly used units of conservation, i.e. ESUs and management units (MUs) [11,12]. An ESU was first defined by Ryder [13] as a population that merit separate management or priority for conservation because of high distinctiveness (both genetic and ecological). This definition was later criticized by Moritz [14] because the term ‘high distinctiveness’ was too vague. He suggested that ESUs should be identified by being reciprocally monophyletic for mtDNA haplotypes and show significant differentiation of allele frequencies at nuclear loci [14]. Moritz also used the term ‘MUs’ which is a less stringent term to accommodate subdivided populations where divergence time has not been sufficient to accumulate evolutionary diagnostic characters. Although Moritz’s paradigm provides a relatively straightforward method of assigning conservation status, this approach has been criticized for a number of limitations [11, 15, 16]. Subsequently, Crandall et al. [15] suggested a different definition and advocated that species designation criteria of ecological and genetic exchangeability may provide a more appropriate means of identifying units for conservation because it takes into account both historical and ecological factors and promotes the maintenance of meaningful adaptive diversity. This approach (i.e. genetic and ecological distinctiveness) has been used to investigate the genetic status of natural populations and recognize different units of conservation in several taxa, such as the Bornean elephant [17], sky-island rattlesnake [18], Giant panda [19], and Bryde’s whale [20]. Moreover, Fraser & Bernanchez [11] have proposed an ESU concept in the aim of proposing a more unified concept reconciling opposing views. They provided a context-based framework called adaptive evolutionary conservation (AEC), in which they suggested that differing criteria will work more dynamically than others and can be used alone or in combination depending on the situation.

Previous genetic studies on mainland Bengal tigers have evaluated tiger populations from different landscape complexes (or geographic regions) in India, such as North, northwest, central, South and northeast, and found moderate to high levels of genetic diversity [21, 22, 23, 24, 25, 26, 27, 28]. Some of the studies have also investigated Bengal tiger population structure and reported high genetic differentiation between tiger populations from the North, central, western and northeast parts of India [21, 22, 23]. However, none of them have investigated tigers from Sundarbans. Genetic investigations may provide insight into the likelihood for persistence of this population, which is currently not connected to any other tiger population from mainland and can contribute to their conservation management [15]. Therefore, in this study, we applied mitochondrial and microsatellite markers to assess the phylogenetic distinctness of tigers in Sundarbans and document the evolutionary relationships among Bengal tiger populations of India. This was done by analysing samples collected from mainland tiger populations in the North and peninsular part of India. Extending our analysis to include populations from different regions also provide a much broader bio-geographical framework within which to interpret data on the isolated tiger population of Sundarbans.

Our ultimate goal was to use a multi-population comparative approach to: (i) determine whether the tigers in Sundarbans have reduced levels of genetic variability relative to other populations of mainland tigers, as might be expected given their small contemporary population size and insular distribution, (ii) estimate the degree of genetic differentiation, historical and recent migration, (iii) determine time since separation from the mainland and effective population sizes of Sundarbans and mainland tiger populations, (iv) evaluate the traits to determine the exchangeability or adaptive differences among tiger populations, and (v) discuss whether Sundarbans tiger population should be considered as a separate ESU and MU, and implications for their conservation or management.

## Material and Methods

### Ethics statement

All tiger samples (blood and tissue) used in this study were provided by forest department of Sundarbans tiger reserve during the routine monitoring, translocations and radio collaring of the conflicted animal. Hence, sample collection did not require any extra handling of the animals. All necessary permissions to get the samples to the National wildlife reference sample repository at Wildlife Institute of India (WII) were obtained from the Field Director of the respective forest reserve (letters no. 2509/WL/2W-296,2(4)/SBR/C-208/09, 401(4)/SBR/C-172/10, 1906/FD/2M-96/06 and 4439/WL/2W/567/06).

### Study area and sampling

**Fig 1:**
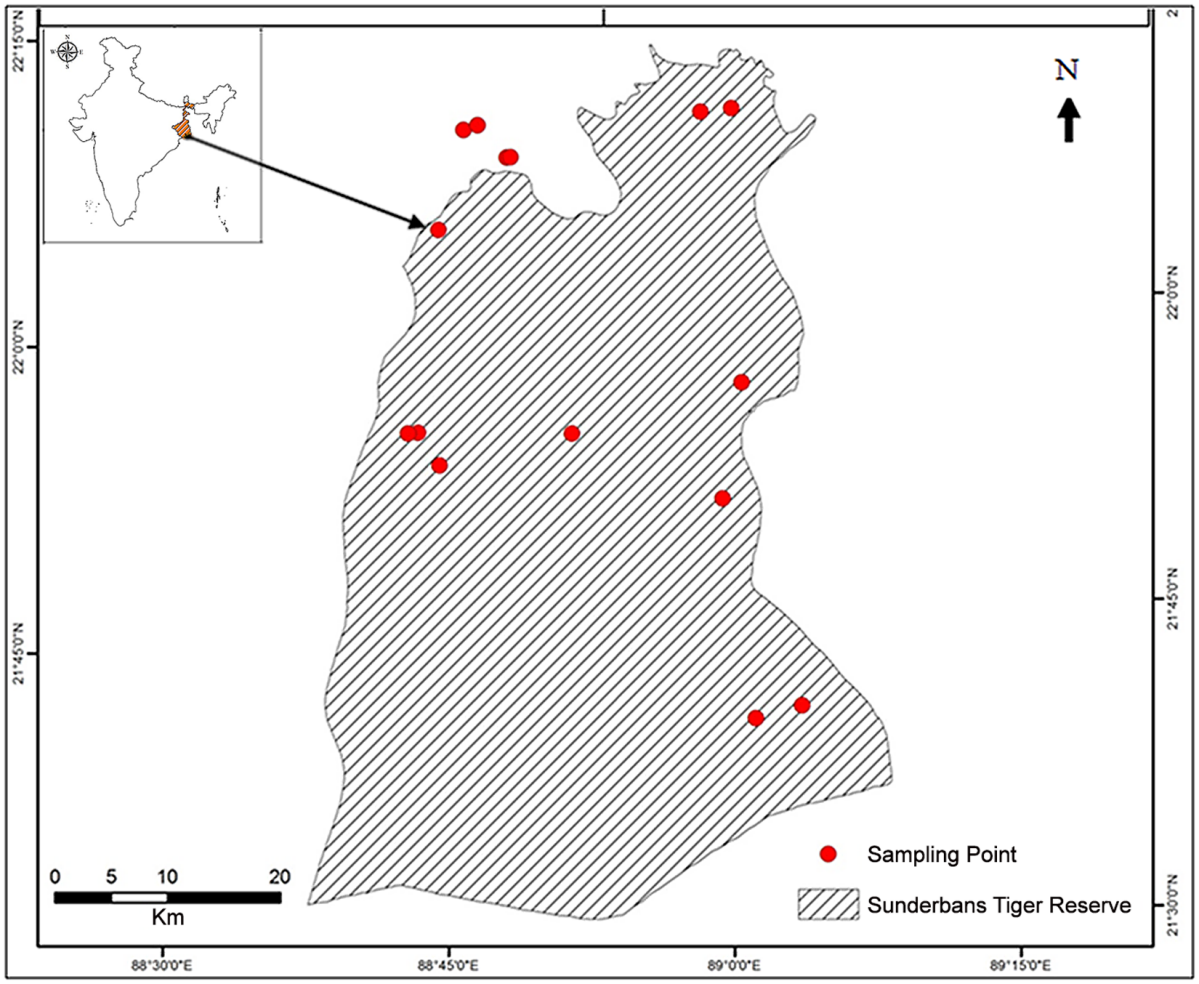
Map showing the location of tiger samples collected from Sundarbans.

In order to adequately assess genetic status of Sundarbans tigers, it is critical to obtain as many biological samples as possible. However, because most of the area in this tiger habitat is inaccessible swamp, it is difficult to non-invasively collect samples, such as feces or hair. We overcome this problem by using opportunistically collected samples by the forest officials. We obtained sixteen tiger samples: blood (*n* = 5), tissue (*n* = 1), hair (*n* = 1), and scat (*n* = 9) from the forest department of Sundarbans tiger reserve (hereafter Sundarbans) (Fig. 1). In order to make appropriate comparison with other mainland tiger populations, we also obtained tiger samples (*n* = 73; scat) from the tiger reserves located in the Central and Eastern Ghats of India (Kanha, Pench, Bandhavgarh, Panna, and Palamau), and (*n* = 62; tissue, blood and scat) from the tiger reserves located in northern India (Corbett, Rajaji National Park and Dudhwa). We pooled all the tiger samples from the Central and Eastern Ghats, and are referred to as “Peninsular India”.

### DNA extraction

Genomic DNA from blood spots stored on FTA Classic cards (Whatman^TM^) was extracted following Smith and Burgoyne [29]. DNA from hairs and tissue samples were extracted using Qiagen DNeasy blood and tissue kit (QIAGEN, Germany) following the instruction of the manufacturer with slight modifications. For scat samples, QIAamp DNA Stool Mini Kit (QIAGEN, Germany) was used. All DNA extractions were performed in a separate space in laboratory dedicated for fecal samples and a negative control was always included to monitor possible contamination. The extracted DNA from each sample was genotyped using two different markers: a) mitochondrial DNA (mtDNA), and b) microsatellites.

### PCR amplification and sequencing of mitochondrial genes

In order to check for the existence of new haplotypes in the tiger samples from Sundarbans, we amplified and sequenced four mtDNA fragments comprising *ND2* (960 bp), *ND5* (1139 bp), *ND6* (443 bp), and *cytb* (555 bp) regions. Fragments were chosen based on previously sequenced regions of other tiger subspecies (including Bengal tiger) that were established as variable and informative, and correspond to that used by Luo et al. [2] and Mondol et al. [22]. Amplification reactions were performed on an ABI 9600 Fast (Applied Biosystem, Switzerland) thermocycler in a total volume of 10 μl containing 1 μl of extracted DNA, 1× PCR buffer, 2.5 mM of MgCl_2_, 200 μM of each dNTP, 1× BSA, 0.5 U of Taq DNA polymerase (Applied Biosystem), and 0.2 μM of each primer. PCR amplification conditions were the same as reported by Luo et al. [2]. All amplified PCR products were analyzed using an ABI 3130 Genetic Analyzer (Applied Biosystem, USA). The quality of the DNA sequences was determined using the Sequence Analysis software package (Applied Biosystem) and validated using SEQUENCHER 4.7 (Gene Codes Corporation, USA). All contigs were manually inspected and were compared with published tiger sequences from other studies.

### PCR amplification and nuclear microsatellite genotyping

Nine microsatellite loci, of which four loci were originally developed for Bengal tiger (PttA2, PttA4, PttC6, PttE5) [30], two (Pun82 and Pun327) for Snow leopard [31] and three (F41, Fca272 and Fca304) for domestic cat [32] were selected for the present study on the basis of small amplicon size, high amplification success and low genotyping error rate [33, 34]. All PCR amplifications were performed as per Mishra et al. [33, 34]. All forward primers were fluorescently labelled. PCR products were analyzed using the ABI 3130 Genetic Analyzer (Applied Biosystem, USA) and alleles were scored manually with GeneMapper 3.7 (Applied Biosystem, USA).

## Data analyses

### mtDNA

In order to examine the phylogenetic relationships among haplotypes, all published tiger mtDNA sequences (including all extant tiger subspecies) were retrieved from GenBank and compared with CLUSTALW in BioEdit, and then manually edited to achieve the best alignment [35]. The sequences used include the tiger mtDNA sequences published by Luo et al. [2] (*n* = 25) and Mondol et al. [22] (*n* = 21). The GenBank accession numbers of mtDNA sequences used are Cytb: AY736634-AY736658, EU661630-EU661650, ND2: AY736684-AY736733, EU661651-EU661671, ND5: AY736734-AY736783, EU661672-EU661691 & FJ228452, and ND6: AY736784-AY736808. Since the length of the mtDNA sequences analysed in the two studies (Luo et al. [2] and Mondol et al. [22]) were not the same, we created two independent datasets and analysed them separately. In total, we analysed 2600 bp of mtDNA sequence with Luo et al. [2] and 1063 bp with Mondol et al. [22] study. Genetic diversity indices, such as gene diversity (*h*), number of segregating sites (*S*), mean number of pairwise nucleotide differences, or nucleotide diversity (*π*) were estimated with 1063 bp mtDNA sequence (*ND2*, *ND5* and *cytb*) using DnaSP 5.0 [36].

The median-joining networks (MJ Network) were constructed with Network 4.6.1.1 [37] by using the median joining algorithm with default settings (weight = 10 and e = 0). In comparison to conventional phylogenetic tree, haplotypic network gives improved relationship when recent multi-furcations occur, the level of haplotype divergence is low, and/or ancestral and derived haplotypes coexist [38].

The program ModelGenerator was used to determine the most appropriate model of substitution [39]. ModelGenerator selects best fit model under the Akaike Information Criterion (AIC) [40]. The chosen model is the one that minimizes the Kullback-Leibler distance between the model and the truth [41]. Based on the chosen alignment, the HKY model of substitution with a discrete gamma model of rate heterogeneity was selected as the most appropriate model for both sequence datasets. For mtDNA, pairwise *F*_ST_ values based on haplotype frequencies were estimated using Arlequin 3.5.1.2 [42, 43].

### Microsatellites

#### Genotyping error and data validation

To assess error rates for genotypes derived from fecal and tissue samples, we randomly selected 10 fecal samples from the field (from Kanha tiger Reserve) and 10 tissue (from Corbett tiger reserve) samples and reanalyzed them (three times for tissue samples and four times with fecal samples). This was done to calculate allelic drop out (ADO) and false allele (FA) error rates using PEDANT 1.0 involving 10,000 search steps for enumeration of per allele error rates [44]. We performed repeated genotyping for all samples and loci to achieve consensus genotypes (minimum two successful attempts). Typographic error assessment and genotyping error due to null allele was assessed using MICRO-CHECKER 2.2.3 [45].

#### Genetic diversity and population structure

Number of alleles (*N*a), mean number of alleles (*MNA*), expected (*H*e) and observed (*H*o) heterozygosity for the nine microsatellite markers were estimated with FSTAT 2.9.3.2 [46]. Allelic richness (*A*_R_) was adjusted for discrepancies in sample size by incorporating a rarefaction method, and was estimated for each site using FSTAT 2.9.3.2 [46]. Hardy-Weinberg Equilibrium (HWE) using exact test [47] and linkage disequilibrium (LD) among all pairs of the microsatellite loci were estimated using GENEPOP 1.2 [48]. We calculated Wright’s *F-*statistics for microsatellite dataset according to the method of Weir and Cockerham [42] and their significance was tested with 10,000 permutations using ARLEQUIN 3.5 [43]. We also used ARLEQUIN 3.5 to perform a hierarchical analysis of molecular variance [43] to determine partition of genetic variation between sampling sites and when grouped by geographic locations.

We used SPAGeDi [49] for an allele-size permutation test (1000 iterations) [50] to assess whether differences in microsatellite allele size (mutation) contributed to genetic divergence (*R*_ST_ > *F*_ST_) or whether divergence is attributable to genetic drift only. We applied also this analysis to the genotyped data for the tiger populations in Peninsular (*n* = 73) and northern India (*n* = 62). The principle of this test is to compare the *R*_ST_ value with the distribution of *R*_ST_ values (called *pR*_ST_) obtained after 1000 random permutations of allele size among allelic states. The observed *R*_ST_ values significantly larger than the *pR*_ST_ indicate that stepwise mutations contributed to the genetic differentiation among subpopulations. Exact tests of population differentiation among the populations were conducted as described by Raymond and Rousset [48] in ARLEQUIN 3.5 [43].

To identify possible distinct genetic clusters and to assign individuals to these clusters, we utilized the Bayesian clustering approach implemented in software STRUCTURE 2.3.3. [51]. The software applies a Bayesian clustering algorithm to identify subpopulations, assign individuals to them, and estimate the population allele frequencies. STRUCTURE sorts individuals into *K* clusters, according to their genetic similarity. The Bayesian clustering analyses were done both with and without prior knowledge of sampling locations (with and without LOCPRIOR) [51, 52]. We analyzed our data by using the admixed model and correlated allele frequencies option to carry out 25 independent runs for each value of *K* between 1 and 10 with a burn-in period of 50,000 iterations and collected data for 500,000 iterations. The most likely value of *K* was assessed by comparing the likelihood of the data for different values of *K* and by the rate of change in the log probability of the data between successive *K* values (Delta *K)* [53]. We ran STRUCTURE using dataset comprised of samples from i) northern India and Sundarbans, ii) Peninsular and Sundarbans iii) northern, Peninsular and Sundarbans, and iv) Palamau (nearest to Sundarbans) and Sundarbans. Structure Harvester [54] was used to estimate and plot Delta *K* [53]. Assignment of individuals to the inferred clusters was estimated according to the highest q-values (probability of membership). STRUCTURE results were visualized with the program DISTRUCT [55].

We also used a spatially explicit clustering method to identify possible genetic clusters as implemented in the program TESS 2.3 [56] that builds a spatial individual neighborhood network using the Voronoi tessellation. The prior distribution of cluster labels is calculated using hierarchical mixture models. It uses the spatial information along with multilocus genotypic data from individuals to define population structure without using predefined population information. TESS 2.3 was run using the both admixture models (CAR and BYM) with spatial interaction parameter set at 0.6, as recommended by Chen et al. [56]. We ran the TESS analysis for 30,000 burn-ins followed by 50,000 run-in sweeps for *K* 2–10. The preferred *K* was selected by comparing the individual assignment results and the deviance information criterion (DIC) for each *K* [57]**.** DIC values averaged over 100 independent iterations were plotted against *K*, and the most likely value of *K* was selected by visually assessing the point at which DIC first reached a plateau and the number of clusters to which individuals were proportionally assigned.

#### Effective population size (Ne)

We estimated short-term effective population size (*N*e) for different tiger populations (northern, Peninsular, and Sundarbans) from genetic data using the approach based on linkage disequilibrium (LD) as implemented in LDNe 1.31 [58]. We used the criterion *P*_crit_ = 0.02, which generally provides a good balance between precision and bias from rare alleles [59]. The analysis was repeated after removing alleles with frequencies (*P*_crit_) lower than 0.01 and 0.05.

#### Gene flow between populations

We estimated the number of migrants between all pairs of sample sites using GENECLASS 2.0 [60]. A Bayesian approach as described by Rannala and Mountain [61] was used along with a resampling algorithm of Paetkau et al. [62] for likelihood computation (L_home_/L_max_), with 1000 simulations at an assignment threshold (alpha) of 0.01.

BayesAss1.3 programme [63] was used to estimate the magnitude and direction of contemporary (past few generations) gene flow among populations. BayesAss 1.3 uses a MCMC algorithm to estimate the posterior probability distribution of the proportion of migrants from one population to another (M) without assuming genetic equilibrium. We took contemporary timescale to be about five to seven generations (i.e. 25–35 years), assuming a generation time of 5 years for tigers [64]. We used 9×10^6^ iterations, with a burn-in of 10^6^ iterations, and a sampling frequency of 2000 to ensure that the model's starting parameters were sufficiently randomized (confirmed by checking changes in likelihood values). Delta values were adjusted to optimize terminal proposed changes between chains (40–60% of the total iterations) to ensure sufficient parameter space was searched [50]. BayesAss1.3 provides mean and 95% confidence intervals (CI) expected for uninformative data that can be used to assess the reliability of data and also identifies first- and second-generation migrants in a population.

#### Sex biased movement

In order to infer whether dispersal between tiger populations (Peninsular, northern, and Sundarbans) is sex-biased, we estimated the relative amount of male and female gene flow following the approach of Hedrick et al. [65]. We first estimated the amount of male differentiation using equation 7a in Hedrick et al. [65].

$$F_{ST(m)}=(2F_{ST}\times F_{ST(f)})÷(F_{ST(f)}-2F_{ST}+3F_{ST}\times F_{ST(f)})$$

and then assessed the ratio of male and female gene flow rates based using equation 7b in Hedrick et al. [65]

$$m_{m}/m_{f}=\;(F_{ST(f)}(1-F_{ST(m)})/F_{ST(m)}(1-F_{ST(f)}).$$

### Demographic analyses

Demographic analyses was performed to calculate the divergence time and population history of Sundarbans tiger by using different molecular markers (mitochondrial and microsatellite). Bayesian skyline plots [66] of mitochondrial effective population sizes through time were produced from the sequences of *ND2*, *ND5* and *cytb* genes of Sundarbans tigers with sequences of northern and peninsular populations. To estimate divergence times and population history, we also used the coalescent-based approximate Bayesian computation (ABC) algorithm in the program DIY-ABC [67] with the microsatellite data of Sundarbans, Peninsular and northern tiger populations (S1 File).

### Ecological exchangeability

In order to investigate if Sundarbans tigers have characteristics that distinguish them from other mainland tiger populations, we examined a large sample of the published literature in order to find evidence of adaptive differences or traits, such as morphology, habitat type, size of prey, competition with other predators, tiger density [5, 8, 10, 68–71].

## Results

Large sized fragments of the mtDNA for at least one fragment of the four mitochondrial genes (*ND*2, *ND*5, Cytb, and *ND*6) were successfully sequenced only from six Sundarbans samples. However, this was not the case with the nuclear DNA amplification. In case of microsatellite markers, out of the 16 samples genotyped, three (two scat and one liver tissue) did not yield results and 13 unique genotypes were identified in the Sundarbans samples. Therefore, mtDNA and microsatellite data from six and thirteen Sundarbans samples were used in further analyses.

### Genotyping error and data validation

Out of all samples, tissue samples did not show allelic drop out (ADO) and false alleles (FA), however, the fecal-extracted DNA showed that ADO ranged between 0 and 11% and FA between 0 and 4%. Genotyping error rate also varied among loci (Table 1). Only two loci (F41 and F327) from Sundarbans were susceptible of containing high frequencies of null alleles (>10%), as identified by Microchecker. However, high frequencies (>10%) were suggested in several loci from Peninsular (PttA4, PttE5 Fca304, Fac272, F41, PUN327, PUN82) and northern (PttA2, PttE5, PttC6, F41, PUN327, PUN82) populations. We also checked for null alleles within individual sampling locations from Northern and Peninsular tiger populations and found indications that several loci have null alleles (S1 Table), but this was not consistent over different sampling sites.

**Table 1:** Allele size range, Number of alleles (*Na*) and genotyping error rates (ADO = allelic dropout, FA = False allele) at nine microsatellite loci with field collected scat (n = 10; for Kanha tiger reserve), tissue samples (n = 10; from Corbett tiger reserve) and scat & hair samples from Sundarbans (n = 8) of wild Bengal tiger.

	Scat (N = 10)				Tissue (N = 10)				Sundarbans (n = 8) scat & hairs			
Loci	Product size range (bp)	Na	ADO (%)	FA (%)	Product size range (bp)	Na	ADO (%)	FA (%)	Product size range (bp)	Na	ADO (%)	FA (%)
PttA2	186–190	3	5	0	188–190	2	0	0	188–196	3	7	0
PttA4	139–145	4	7	0	145–153	4	0	0	143–151	3	3	1
PttC6	172–176	3	0	2	174–178	3	0	0	174–178	3	0	0
PttE5	183–187	2	4	0	174–190	4	0	0	182–192	3	8	2
FCA304	122–142	3	0	0	122–128	4	0	0	122–126	3	1	0
FCA272	115–121	4	11	0	119–127	5	0	0	117–123	4	1	0
F41	171–187	4	8	0	171–179	3	0	0	171–191	4	5	0
PUN327	88–96	5	0	4	84–90	3	0	0	84–96	4	1	0
PUN82	111–115	3	0	4	101–119	5	0	0	114–118	3	5	2

### Genetic diversity and population structure

**Fig 2:**
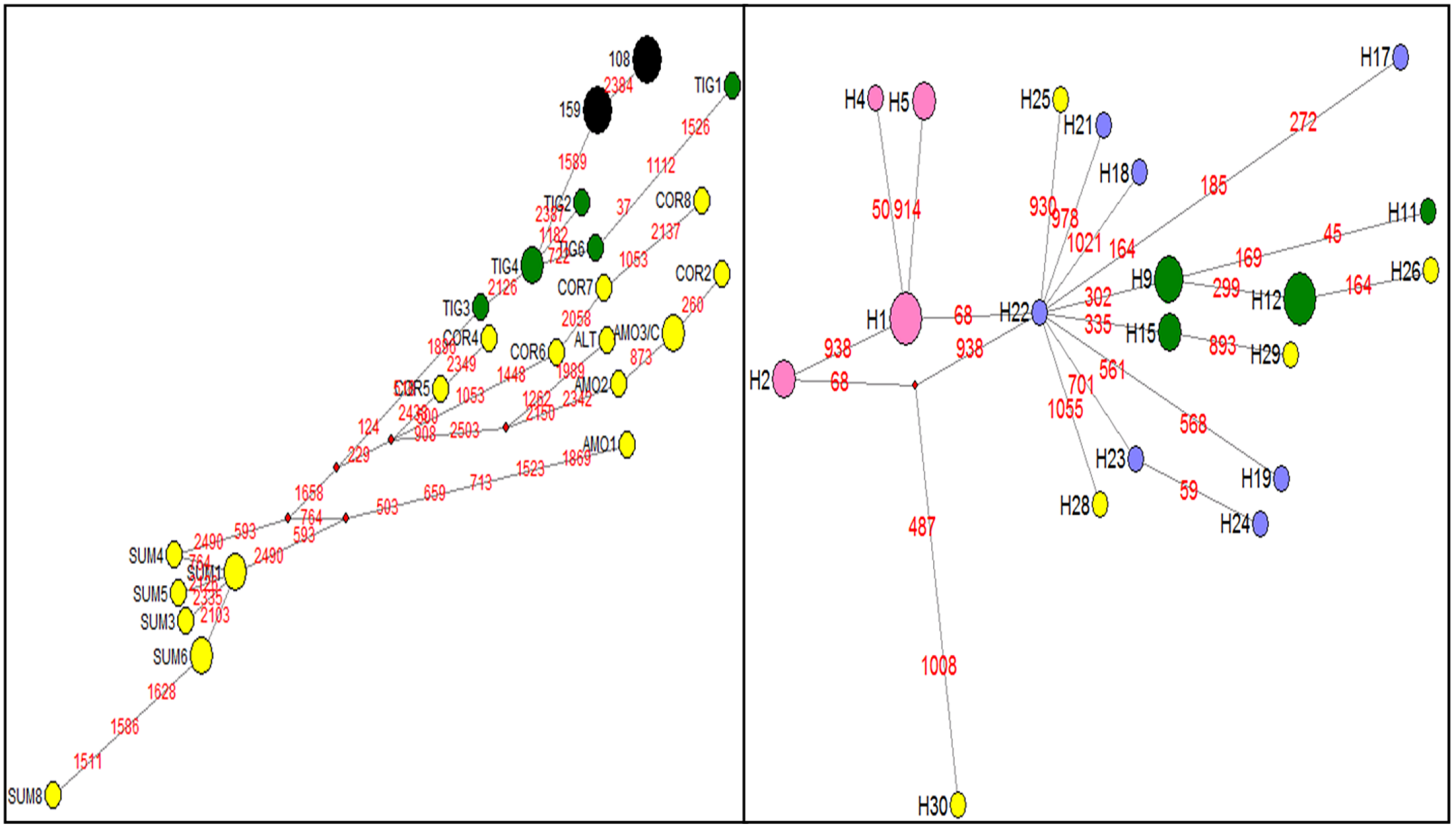
Median-joining network created from four mtDNA genes (*cytb*, *ND2*, *ND5* and *ND6*) (in total, 2600 bp) depicting genetic relationship between all haplotypes found in tigers. (a) haplotypes found in Sundarbans tigers (in black) and all other six tiger subspecies (in yellow and green color, from Luo et al. 2004) [2], (b) all haplotypes found in Bengal tiger populations from this study and Mondol et al. [22]. Pink: North India, Yellow: Central India, Blue: South India, and Green: Sundarbans. The sizes of the circles are proportional to the haplotype frequencies.

Comparison of 2600 bp mitochondrial sequences of the Sundarbans tigers with the sequences from six other tiger subspecies revealed that Sundarbans tigers shared three nucleotide substitutions that were identified as specific (or diagnostic) to Bengal tiger subspecies. The mtDNA amplification target includes 13 single nucleotide polymorphisms (SNPs), of which 3 are diagnostic (fixed differences) for *P*. *t*. *tigris* [2]. In addition, we found two new haplotypes that were shared only among Sundarbans tigers. One variable site was observed at *ND5* region and was shared among all six Sundarbans samples. However, the other variable site at *cytb* region was found only in three samples. These substitutions differentiated Sundarbans tigers from rest of the other tiger subspecies. In median joining network, the Sundarbans haplotypes composed a monophyletic sister group with other Bengal tigers (Fig. 2).

Further comparison of Sundarbans tiger haplotypes with the other Bengal tiger sequences obtained from northern and Peninsular tiger populations revealed that these haplotypes are specific to Sundarbans samples and there were no other shared haplotypes between the samples. Interestingly, one of the two Sundarbans haplotypes observed in the present study corresponds to the unique haplotype (TIG29) as previously reported by Mondol et al. [22]. The study by Mondol et al. [22] included two tiger samples from Sundarbans and reported two different haplotypes (TIG23 and TIG29), which were based on substitution in the conserved *cytb* region. However, the authors did not analyse the complete *ND5* region from their samples from Sundarbans. Therefore, the other haplotype (at *ND5* region) is reported here for the first time. This new haplotype have been submitted to GenBank under accession numbers JX074818-JX074823. We also compared mtDNA sequence data (*ND5*, *ND2*, *ND6* and *Cytb* regions) of tigers from Northeast tiger populations (Kaziranga Tiger Reserve, Manas National Park, Pakke Tiger Reserve, Twai wildlife sanctuary, and Orang National Park) that were published in Sharma et al. 2008, 2010 [21, 23] and Mondol et al. 2008 [22]. We did not find any shared haplotype between Sundarbans and adjoining areas (northeast and Brahmaputra flood plains). We also analyzed two samples from Debang wildlife sanctuary (located in the Brahmaputra flood plains) for the presence of Sundarbans haplotype), but the observed haplotypes were completely different.

Molecular diversity indices such as haplotype (*h*), nucleotide (π) diversity was higher in the Peninsular India tiger populations than in the Sundarbans (Table.2). The values of *h* and π varied among populations, ranging from 0.679 to 1.00 and 0.001 to 0.002, respectively. Samples from Sundarbans and northern population exhibited almost similar *h* and π values (Table 2).

**Table 2:** Genetic diversity indices and effective population size (*N*e) of Sundarbans, Peninsular, and northern India tiger populations based on nine microsatellite markers and partial mtDNA sequence.

Tiger population	Microsatellites							Mitochondrial DNA			
	N	MNA	AR	Ho	He	FIS	Ne (95% CIs)	N	S	h	π
**Sundarbans**	13	3.33	3.24	0.491	0.587	0.109	-242.6 (102Infinite)	8	3	0.679	0.001
**Peninsular**	73	7.33	4.80	0.492	0.707	0.300	73.3 (48.4–128.4)	15	19	1.00	0.002
**Northern**	62	4.88	4.18	0.401	0.674	0.394	47.9 (29.5–92.3)	8	3	0.82	0.001

*N*: number of samples, *MNA*: mean number of alleles, *A*_*R*_: allelic richness, *Ho*: observed heterozygosity, *He*: expected heterozygosity, *F*_*IS*_: Wright’s fixation index estimates, *N*e: effective population size, *S*: number of segregating site, *h*: Haplotype diversity, and π: Nucleotide diversity.

All nine microsatellites were polymorphic, with between 3 and 4 alleles per locus in the Sundarbans tiger samples. The mean number of alleles (*MNA*) for Sundarbans tigers was 3.33, which is lower than in Peninsular and northern India tiger populations (7.33 and 4.88, respectively). Allelic richness corrected for the sample size was lower in Sundarbans (3.24) than in Peninsular (4.80) and northern India population (4.18) (Table 2). Also, a positive correlation between the mean number of alleles and sample size (*r* = 0.88) was observed. Average expected heterozygosity (*H*e across loci) values were higher in the northern and Peninsular India tiger populations than the Sundarbans and ranged from 0.58 in Sundarbans to 0.70 in Peninsular India populations, whereas average observed heterozygosity (*H*o) ranged from 0.40 in northern to 0.49 in Sundarbans and Peninsular India tigers.

Two loci in Sundarbans, and six loci each in the northern and Peninsular tiger populations were not in HWE. *F*_IS_ values over all loci were significantly different from zero and positive in the northern and Peninsular tiger populations, ranging from 0.109 (Sundarbans) to 0.394 (northern) (Table 2). The most likely explanation for deviation from HWE is the presence of multiple sub-populations within a single population across a broad geographical area in the northern and Peninsular part of India, also known as the Wahlund effect [12, 72].

The allele size permutation test [50] indicated that stepwise mutations (SMM) at microsatellite loci did not significantly contribute to genetic variation among Sundarbans, Peninsular and northern India populations compared to genetic drift and migration (*P* = 0.1125), therefore we used only *F*-statistics.

Using mtDNA, the pairwise *F*_ST_ (PhiST) calculations indicated that all tiger populations were genetically differentiated from each other, with moderate to high *F*_ST_ (PhiST) values ranging between 0.116 and 0.250 (*p* < 0.05; Table 3). For microsatellite data, we found a considerable level of genetic differentiation between Sundarbans and mainland (i.e. northern and peninsular) tiger populations. Table 3 shows that the pairwise *F*_ST_ values ranged from 0.03 to 0.07 (*p* < 0.001). AMOVA test revealed that variation within populations, between populations, and among regions accounted for 90.12%, 9.40%, and 0.47% (*p* < 0.001) of the total variation, respectively.

**Table 3:** Pairwise *F*st values between Sundarbans, Peninsular and northern India tiger populations using microsatellite (below diagonal) and mtDNA markers (above diagonal).

Bengal tiger populations	northern	Peninsular	Sundarbans
**Northern**	0	0.058*	0.250*
**Peninsular**	0.030 **	0	0.116*
**Sundarbans**	0.070 **	0.069*	0

*p < 0.05

** *p* < 0.001

**Fig 3:**
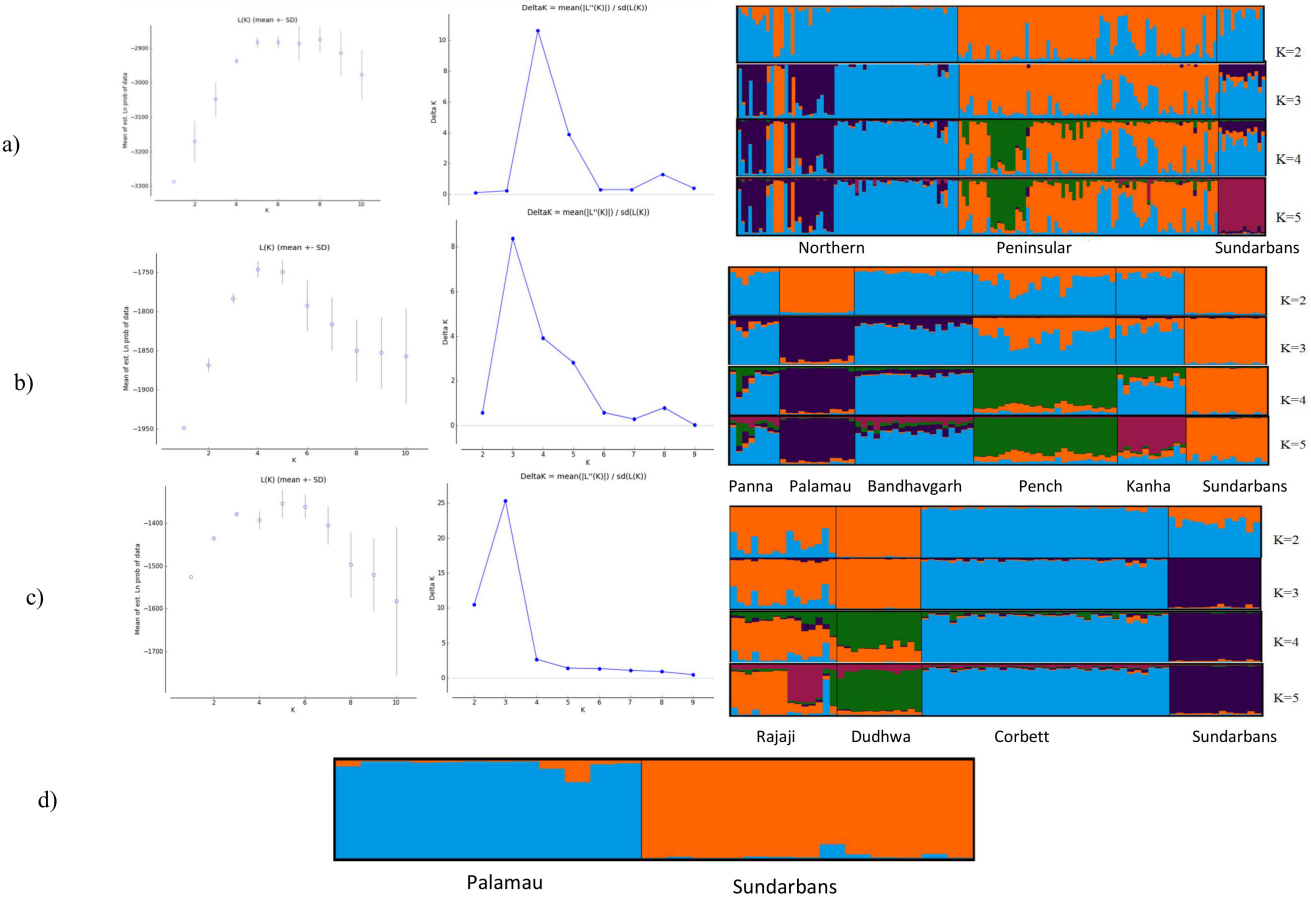
Individual assignment probabilities of Bengal tiger populations analyzed using the model-based program STRUCTURE run of K = 2 to 5 for different dataset/tiger populations (See methods for details): a) northern, Peninsular and Sundarbans, b) Peninsular and Sundarbans, c) northern and Sundarbans and d) Palamau and Sundarbans. Each individual is represented by a vertical bar and indicates the probability of membership in each cluster.

Bayesian cluster analysis of microsatellite genotypes in STRUCTURE suggested the existence of five genetic clusters (mean Ln P (X/K) = -2882.64), when data were analysed across a large geographical scale (i.e., northern + Peninsular + Sundarbans). However, delta *K* analyses [53] suggest that the most likely number of clusters is four. The maximum value for the estimated mean likelihood as inferred by Ln P (X|*K*) was found at *K* = 5. It is observed in many studies that delta *K* does not produce a proper resolution of population structure [73]. Faubet et al. [74] pointed out some problems with Evanno method of delta *K* calculation and there is always a potential of including in the calculation *K*s of several chains that have not converged leading to unreliable results. Based on their observation, Faubet et al. [74] concluded that “for the simple finite island model (that we considered), Evanno et al.’s [53] method does not perform better than the original approach proposed by Pritchard et al. [51]” and provocated the use of the strategy proposed by Pritchard et al. [51]. They suggested that it may not always be possible to know the TRUE value of *K*, but we should aim for the smallest value of *K* that captures the major structure in the data. On the basis of above observations we found maximum assignment at *K* = 5, as indicated by the LnPD. However, for *K* > 2, the likelihood values showed only a slight increase, suggesting the presence of five differentiated populations. Hence, we showed assignment probabilities of the tigers for *K* value at 2 to 5 (Fig. 3). At *K* = 2, the individuals from northern and Sundarbans tiger populations were clustered together in one group and separated from the rest of the peninsular tiger populations, and there was no geographical pattern in the assignment of the tiger individuals. At *K* = 5, all individuals within Sundarbans were strongly assigned together to a separate cluster with high probability (q > 0.8) and there was no evidence of admixture (Fig. 3). Interestingly, the results did not change when STRUCTURE analysis was repeated on the separate data set comprised of populations only from northern or Peninsular regions. STRUCTURE showed further population structure within the northern and Peninsular regions; however, all individuals from Sundarbans were grouped together and strongly assigned to one cluster (q > 0.8, Fig. 3). We also did not find any evidence of migration between Sundarbans and Palamau, and all individuals in both populations were assigned to their original populations.

TESS gave results similar to STRUCTURE *i*.*e*. suggested existence of five genetic clusters under both models (CAR and BYM) using the DIC criterion. In these models, all the individuals from the geographically isolated Sundarbans were grouped together in one cluster both at *K* = 5 and *K* = 6 (S1 Fig).

**Fig 4:**
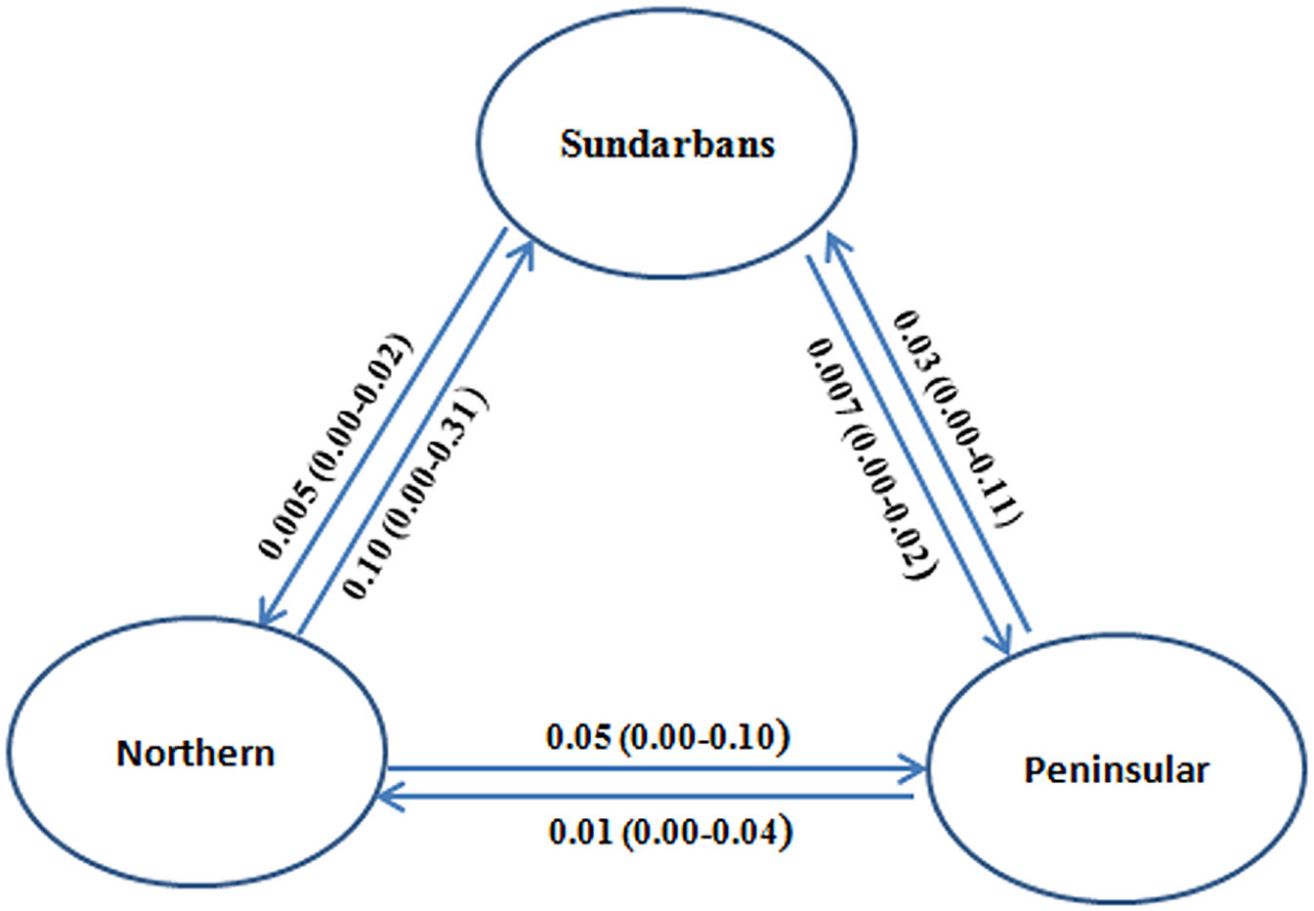
Migration rates detected with BayesAss-program for each population with 95% credible set. Arrows show the direction of gene flow.

We did not detect first generation migrants in Sundarbans population with GENECLASS using a threshold *p*-value of 0.01. However, results from the program BayesAss suggested that low amount of long-term migration may have occurred from the northern and Peninsular India into Sundarbans population (0.10% and 0.03%, respectively; Fig. 4)**.** The 95% credible intervals of these estimates were larger than zero, suggesting significant amount of migration. The estimated migration rates from Sundarbans to northern and Peninsular India were lower (0.005% and 0.007%, respectively) and the lower limit of 95% credible intervals of both estimates were 0, suggesting that there were no migration out of the Sundarbans population. The 95% credible intervals of the estimates from Peninsular and northern into the Sundarbans population overlapped and thus the differences in asymmetric migration rates were not statistically significant.

### Effective population sizes

Estimates of effective population size (*N*e) for Sundarbans samples were negative values and hence, were unattainable using the LD method. This was as a result of the sample size to effective population size ratio being too small and therefore the sampling error overpowered the effect of drift within the sample. However, estimates of *N*e in the Peninsular tiger population (73.3) are higher in comparison to the northern (47.9) India tiger populations (Table 2).

### Sex biased movement

The estimated amount of the genetic differentiation from male gene flow, “*F*_*ST(m)*_” between the Northern and Sundarbans populations was 0.215 which was lower than amount of female differentiation (*F*_*ST(f)*_ = 0.250). The ratio of male to female gene flow between Sundarbans and Northern India was m_m_/m_f =_ 1.21, suggesting slightly higher amount of male than female mediated gene flow between these populations in the past. However, the estimates of male gene flow and ratio of male to female gene flow between Peninsular and Sunderbans populations were not workable using Hedrick et al. [65] equations 7a and 7b (i.e. > 1 for *F*_*ST(m)*_ and negative for m_m_/m_f_) suggesting that all the assumptions of the method were not valid in this case.

### Demographic analyses

**Fig 5:**
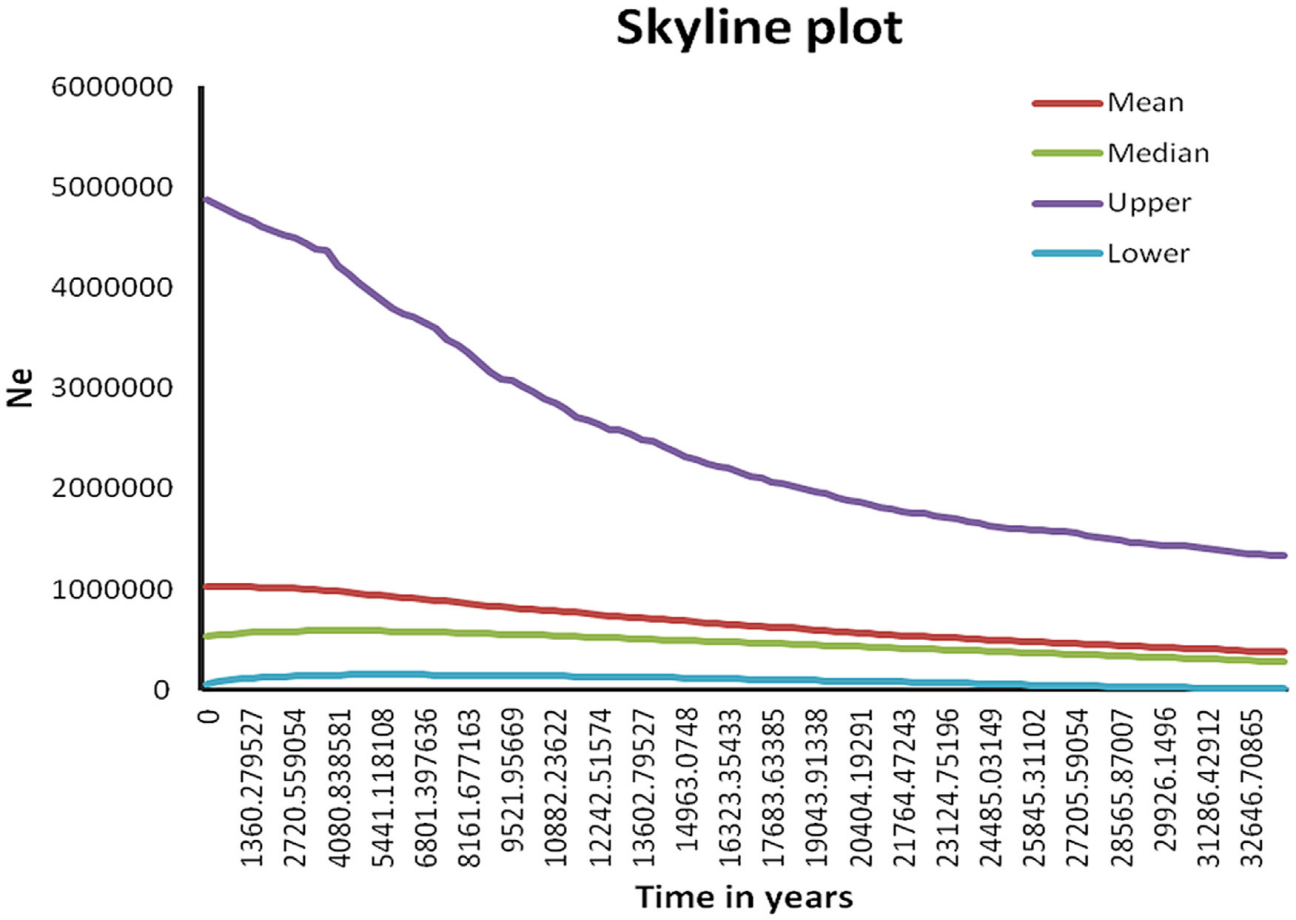
Bayesian skyline plots showing the demographic history of tigers in India.

For the mtDNA, the Bayesian skyline plots revealed slight, but steady growth of the mitochondrial effective size during the last tens of thousands of years. However, the median in the skyline plot shows a decrease of the population size beginning about 1000–2000 years ago (Fig. 5).

**Fig 6:**
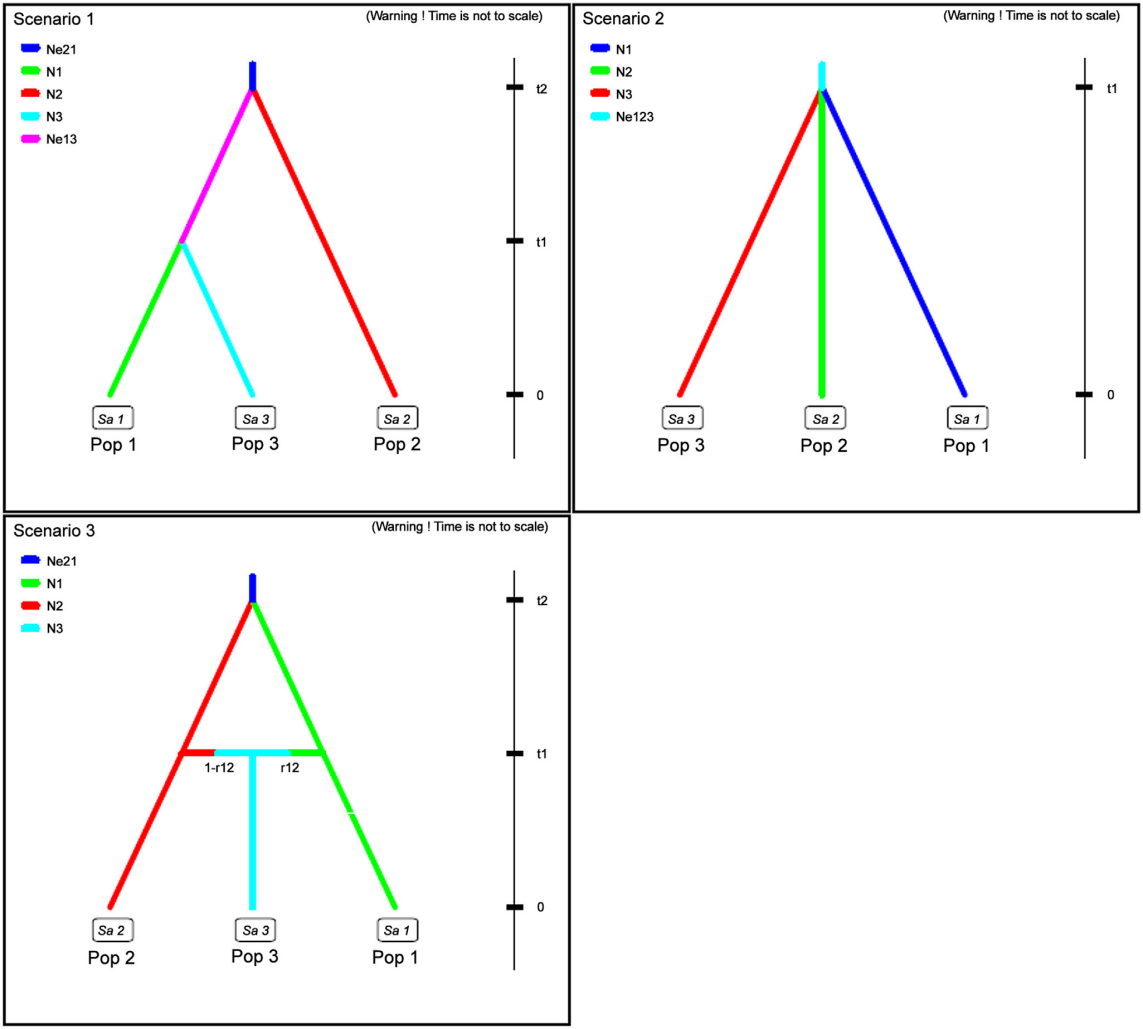
Scenarios tested with DIYABC. Pop 1 = northern India, Pop 2 = Peninsular India and Pop 3 = Sundarbans.

Interestingly, our results from microsatellite data unambiguously point to a scenario similar to the one revealed by mtDNA. The DIYABC analyses suggested that recent divergence explains the observed genetic structure. With the data set divided according to STRUCTURE and TESS, all the explored coalescent scenarios involved estimates of a recent to ancient divergence (Fig. 6). The posterior probability estimates were quite similar among scenario 1 and 2 regardless of the splitting time. In the two most likely scenarios (scenario 1 and 2), the divergence and founding of Sundarbans tiger population was as recent as around 125 and 384 generations ago, i.e. around 600 and 1900 years before present. Estimates of effective population size of present Sundarbans tiger population is around 1000 and similar among the scenarios (Fig. 6, Table 4). The two most likely scenarios (Scenario 1 and 2) showed high posterior probability values using different validation; scenario 1 using direct method (0.5372), whereas scenario 2 through the logistic regression (0.4711). Tests for confidence in scenario choice were not conclusive; type I error (i.e., true scenario did not have the highest probability) for scenario 1 was 0.304 and 0.342 and for scenario 2, 0.164 and 0.264 using direct and logistic regression methods, respectively. Type II error (i.e., the false scenario was not rejected) for scenario 1 was 0.151 and 0.171 and for scenario 2, 0.082 and 0.132 using direct and logistic regression, respectively. One out of 36 statistics simulated according to scenario 1 was significantly smaller than observed (proportion 0.036), whereas none was significantly smaller when simulated according to scenario 2. This indicates slightly more support for scenario 2 over scenario 1, but is not conclusive. Therefore, the parameters were estimated according to both scenarios and also by combining them (scenario 1+2) (Table 4).

**Table 4:** Historic scenarios (1–3) explored with DIYABC to explain present genetic structure in the Bengal tiger populations from northern, Peninsular and Sundarbans.

**Scenario**	*****N***e**				t1	t2
	northern	Peninsular	Sundarbans	Ancient		
**1**	4 410 (1 020–9 510)	21 200 (8 020–47 600)	1 080 (359–8 430)	7 590 (1 840–19 200)	125 (33–1 160)	862 (226–3 770)
**2**	3 020 (927–9 300)	7 270 (3 340–45 100)	1 320 (389–7 940)	11 700 (2 600–18 800)	384 (98.4–1 540)	NA
**1+2**	3 550 (1 030–9 460	19 000 (5 930–47 600)	1 260 (312–7 980)	NA	139 (32.5–1 120)	NA

Posterior probabilities (95% CI) after direct and logistic regression on the 1% simulated data most similar to the observed data, and mode (95% CI) of estimated time since divergence and admixture events (number of generations, t1 and t2).

### Ecological exchangeability

Further assessment of exchangeability using ecological data, such as differences in morphology, habitat type, size of prey species, and competition with other predators revealed that Sundarbans tiger landscape is distinct from other tiger landscapes in India (Table 5). For example, smaller skull size and body weight of Sundarbans tigers in comparison to mainland (i.e. northern and Peninsular) tigers [10] reflects adaptive differences between them and is most likely due to their different ecological niches. In fact, most of the mainland Indian Bengal tiger´s occupy tropical forests [69], able to predate large size prey species (> 50 kg, e.g. Sambar, nilgai and gaur) [70], and compete with sympatric species, such as common leopard. However, tigers in Sundarbans inhabit mangrove forests [68], consume small size prey [8], and there are no competition with other sympatric carnivores [5]. Hemmer et al. [75] suggested that carnivore always strive to have a minimal body mass and size to conquer prey species over certain size. Small body size confers an energetic advantage to Sundarbans tigers and may contribute to their survival in the marshy land [76, 77, 78]. Absence of leopard and low density of tigers in Sundarbans (4.3 tiger per 100 km^2^) in comparison to other landscape (16 tiger per 100 km^2^) [5] reduces inter as well as intraspecific competition. Lack of competion likely to have a strong influence on Sundarbans tiger adaptation to local environmental conditions and allow them to utilize the natural resources.

**Table 5:** Comparison of ecological exchangeability between Sundarbans and mainland Bengal tiger landscape in India.

Ecological exchangeability Parameter	Sundarbans tiger landscape	Mainland Bengal tiger landscape
**Morphology**	Small skull size and body weight of females ~80 kg [10]	Large skull size and body weight of female ~ 160 kg [10]
**Prey Species**	Small size prey (Chital and Wild pig) [8]	Large size prey (Sambar and Nilgai) [69]
**Habitat**	Mangrove forest [68]	Tropical forest [70]
**Competitor**	None [5]	Leopard [5]
**Density**	4.3 tiger per 100 km^2^ [5]	16 tiger per 100 km^2^ [5]

## Discussion

### Genotyping error and data validation

We observed a statistically positive *F*_IS_ for the tiger populations in the northern and Peninsular India, indicating a deviation from HWE. The most likely explanation for deviation from HWE in our study is the presence of multiple sub-populations sampled within a single population across a broad geographical area in the Northern and Peninsular part of India (Wahlund effect) [72, 12]. We found that many loci were out of HWE in the global test, but not consistent when samples were analyzed as separate populations for northern and peninsular India. As suggested by Allendorf and Luikart [79], we also calculated *F*_ST_ for each locus between the subpopulations. As expected, we found that the excess of homozygotes was caused by subdivision; those loci with showed the greatest differentiation between subpopulation also showed excess of homozygosity. Genotypic clustering computer programme STRUCTURE also suggested that there are three to four distinct subpopulations in northern and peninsular tiger population. On the basis of all these observation and detection of many subpopulations in the Northern and Peninsular population concluded that, Wahlund effect was the most probable reason for the HWE deviation.

### Genetic diversity and population structure

The main result of our study is the finding that Sundarbans tiger is not a separate tiger subspecies and should be regarded as Bengal tiger (*P*. *t*. *tigris*) subspecies. MtDNA analyses undertaken first time with 2600 bp on Sundarbans tiger samples revealed the presence of known diagnostic sites (SNPs) for Bengal tiger subspecies and a close phylogenetic relationship of haplotypes found in Bengal tigers [2, 21, 22]. Median-joining Network using *cytb*, *ND2*, *ND5* and *ND6* genes revealed that the haplotypes typical to the Sundarbans tigers comprise a separate group that stems from Peninsular India (Fig. 2) and further groups into two lineages.

Our findings indicate that Sundarbans tiger population contains two new closely related mitochondrial lineages, which had not yet been detected previously. The frequencies of a number of mtDNA lineages in the Sundarbans deviate noticeably from those in mainland tiger populations, suggesting that founder effects and genetic drift may have had a considerable influence on the Sundarbans gene pool, although low sample size may have also biased our results. Analyses of other samples from nearby tiger populations in the mainland, such as northeast India and Brahmaputra flood plains did not reveal Sundarbans haplotype (data not shown). The absence of the haplotype unique to Sundarbans from other tiger populations indicates that practically no emigration of females has taken place from Sundarbans to Peninsular, northern or north east India after geographical isolation.

Our study revealed relatively low levels of genetic variation in Sundarbans tigers, using both mitochondrial sequence and nuclear genetic markers. The levels of genetic variation in Sundarbans tigers (*H*e = 0.59) are lower than that observed in other tiger populations from the mainland (*H*e = 0.67–0.70). The observed heterozygosity of Sundarbans (*Ho* = 0.49) was slightly higher in comparison to northern tiger populations (Table 2), but this could be a biased estimate due to small sample size [80]. Interestingly, the levels of genetic variation found in Sundarbans tigers are still lower than that observed in other mainland Bengal tiger population from other geographical regions, for instance, Northwest and Central India *H*e = 0.70 [25], Central India *H*e = 0.81 [28, 27], and Northeast India *H*e = 0.70 [24]. In assessing diversity estimates from different studies, it should be mentioned that the values are not totally comparable, as different microsatellite have been used.

A closer look at the population genetic pattern inferred from our mitochondrial and microsatellite data analyses suggest that Sundarbans tigers are the most divergent group of Bengal tigers. Both the applied mtDNA and microsatellites demonstrate genetic differentiation and structure between tigers in the different geographical regions, with the Sundarbans being significantly differentiated from the northern and Peninsular populations. Pairwise *F*_ST_ values were significant between tiger populations in all major geographic regions and suggest reduced gene flow between the tiger populations in Sundarbans and mainland. As expected, genetic differentiation between the Sundarbans and mainland tiger populations was stronger for mtDNA (*F*st values between 0.11 and 0.25) than nuclear DNA (*F*st = 0.07). It could be due to strong philopatry, territoriality among females, and low or no immigration in Sundarbans. These levels of genetic differentiation also imply a strong impact of local genetic drift (e.g. fixation and loss of alleles), indicating that the effective population size of Sundarbans population is very small and that current gene flow among them is likely very low. Several factors likely contribute to this common pattern including the larger effective population size of nuclear genes, differences in the rate and mode of mutation, and sex-biased dispersal in tigers [2, 22, 81]. Our estimate of the ratio of male to female gene flow between Sundarbans and Northern India was 1.21 suggesting only slightly higher amount of male than female mediated gene flow. Moreover, the estimates between Peninsular and Sunderbans populations were not rational suggesting and all the assumptions of the method were not valid. Hedrick et al. [65] approach used here assumes that effective population sizes for females and male are equal. However, among Bengal tigers effective population sizes differ substantially because of the variance in male reproductive success is much higher than in females due to differential mortalities [64, 82] and so that the effective number of males is much smaller than the effective number of females. Accordingly, the estimates of the mm/mf ratios are too low. Unfortunately, exact estimates of male and female effective population sizes are not available for Bengal tigers and the estimates could not be corrected.

This finding was also supported by the detection of Sundarbans as a separate population by the Bayesian clustering analysis (STRUCTURE and TESS) for microsatellite markers. These results are not surprising, given the field-based knowledge on the current and historical landscape connectivity between Sundarbans and mainland areas [5, 10]. Further, the high genetic structure observed among these tiger populations studied herein suggests that human disturbance of the landscape is responsible for this difference. Indeed, in the last 600–800 years, very large parts of the forest around Sundarbans have been logged and are under increased anthropogenic pressure [83, 84]. Increasing development has resulted in Sundarbans forests becoming more fragmented, isolated, and reduced in size. At present, it is not known if there is a gene flow between the tiger populations in the Indian and Bangladesh part of Sundarbans. Therefore, further investigation on a large sample size (including samples from both the Indian and Bangladesh part of Sundarbans) is required to understand and confirm their genetic status.

We observed that the microsatellites have considerable allele sharing among different tiger populations. The lack of population specific autosomal markers could mean the tiger populations are not as evolutionary isolated as the mitochondrial phylogeny suggests. However, the complete reciprocal monophyly, high divergence, and geographically structured lineages for the mitochondrial data would also mean that if ongoing gene flow were responsible for allele sharing, migration would have to be strongly male biased. The absence of first generation migrants, high assignment probabilities to the population of origin, and low long-term migration between Peninsular and Sundarbans populations as estimated by the Bayesian methods may suggest sex-biased dispersal between these populations until very recent times.

### Demographic analyses

Interestingly, the demographic analyses using different markers (mtDNA and microsatellites) showed similar results and suggested a recent rather than an ancient divergence of Sundarbans tiger population. Bayesian skyline analysis provided clear evidence of a recent historical reduction in effective population size. These results are supported by Approximate Bayesian analyses (ABC), which suggest that Sundarbans tiger population might have diverged from the mainland tiger population within last 2000 years. The DIYABC estimates involved wide posterior distributions but unanimously suggested that recent divergence explain the observed genetic structure of Bengal tigers in India. ABC analysis suggests high effective population size of Sundarbans (*N*e *=* 1000) and it might be due to the fact that the founder individuals of Sundarbans were with high levels of genetic variation. It is possible because present population of Sundarbans was part of a large contiguous population of Central India (Peninsular) in the recent past. Hence it does appear that the population has still retained the signals of high effective population size of Peninsular India. Indeed Mondol et al. 2009 [22] suggested that the historical effective population size of tiger populations in Peninsular India have been large in the recent past and was around 23,280 (2,964–151,008).

**Fig 7:**
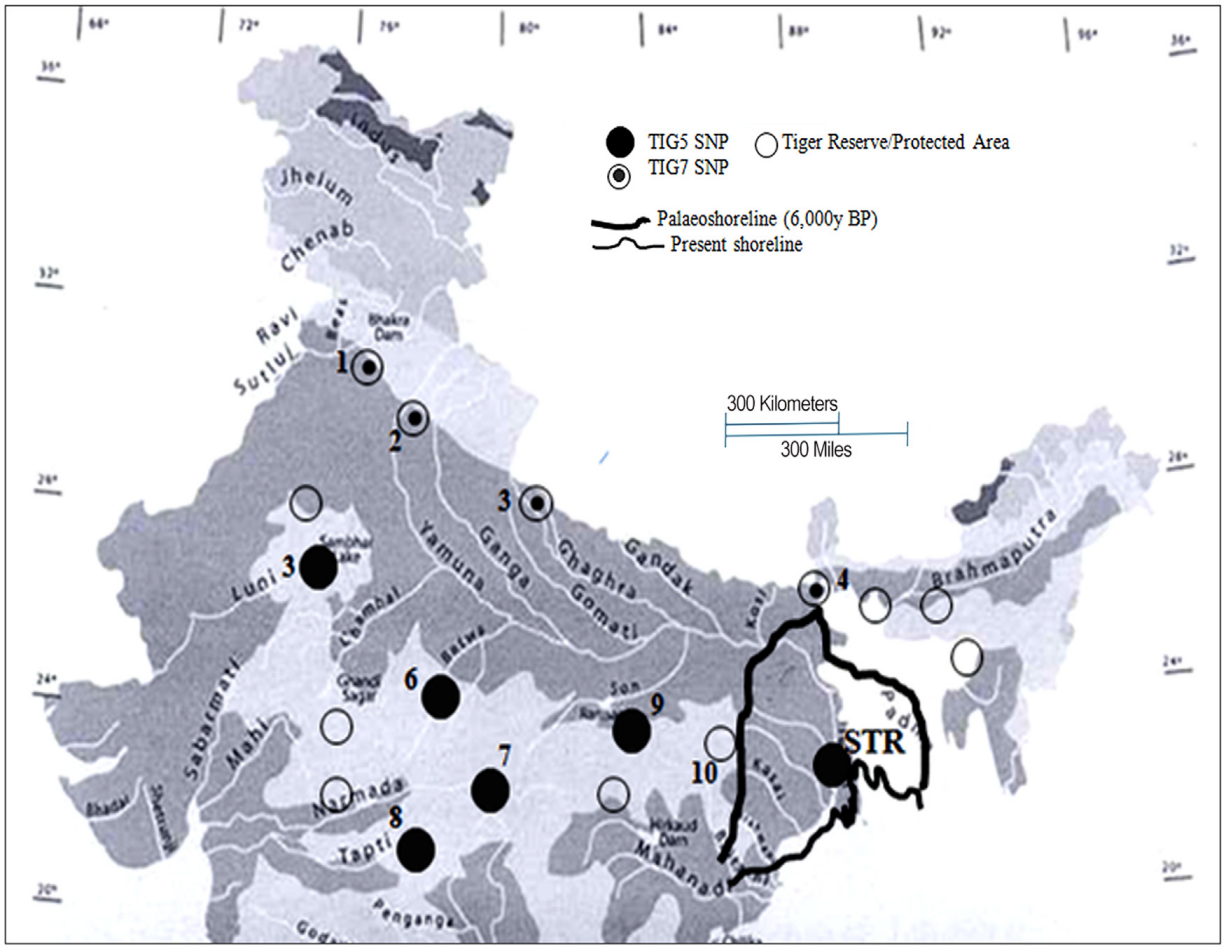
Distribution of TIG5 and TIG7 mitochondrial DNA haplotypes observed in Bengal tiger population and expansion of irrigated agriculture due to the Ganges basin and canals around Sundarbans. (1- Rajaji NP, 2- Corbett TR, 3- Dudhwa TR, 4- Buxa TR, 5- Ranthambhore TR, 6- Bandhavgarh TR, 7- Kanha TR, 8- Pench TR, 9- Palamau TR, 10-Simlipal TR, Sundarbans- Sundarbans Tiger Reserve).

It is striking that for both marker types the data point towards a contraction that took place in the last 2000 years and that is difficult to explain by recent anthropogenic activities alone. Several independent lines of evidence indicate that series of climatic changes profoundly influenced the geography and vegetation in many parts of Sundarbans, leading to shifts in the extension and distribution of different habitat types. These biogeographical changes are thought to have had a profound impact on the geographic distribution of the Sundarbans fauna, including tigers [85]. During the mid-Holocene warm period (around 6,000 years before present) the shoreline of North-east India Peninsula was located in the west relatively close to the foothills of the Himalaya [86] (Fig. 7). Accordingly, the present Sundarbans region was not available for terrestrial life until the shoreline moved eastward and tigers could colonize Sundarbans from the mainland, i.e. peninsular parts. Later, a shift in the course of river Ganges brought changes in the agricultural land use in the areas adjoining the Sundarbans [87]. The anthropogenic activities have taken place in the late 17^th^ century, such as conversion of forest areas to cultivated land, establishment of historical trading places [84], and extension of agriculture due to development of irrigation canal in the Ganges basin. This has resulted in increased human population density (1437.4 person /km^2^), and settlement of people in and around Sundarbans [88]. Severe habitat fragmentation and habitat loss caused complete isolation of tigers in Sundarbans because they are very sensitive to human disturbance [89, 4].

The recent deforestation in the last couple of centuries has most certainly affected the Sundarbans tigers, though we may not be able to detect and date such very recent factors. Indeed, it is also possible that all these ancient climatic and recent anthropogenic factors combined have shaped the history of present day Indian tiger populations, including the Sundarbans [22,26, 28]. Genetic data from a more diverse array of co-distributed megafauna from Sundarbans mangrove ecosystem should provide a comparative framework and depict a clearer picture of Sundarbans tiger population dynamics to better explain their response to climate change and human activities.

### Ecological exchangeability

Consequences of the level of ecological exchangeability from other Bengal tigers reinforce the distinctiveness of Sundarbans tiger population, and suggest that morphological and behavioral changes in Sundarbans tigers might be adaptations to new mangrove habitat and availability of small-sized prey (Table 5). Based on the morphological variations, Barlows et al. [10] concluded that Sunderban tigers are in early stage of allopatric speciation. The divergence during allopatric speciation leads to reproductive isolation (a factor for ESU) [90, 91] but it is a time taking process in mammals (around 2–4 million years) [92, 93]. We inferred a very recent demographic event in Sundarbans tiger population and given sufficient time, this may lead to reproductive isolation of Sundarbans tigers. Hence, historical evidences and genetic data obtained in this study also support the fact that Sundarbans tiger is in the early stages of allopatric speciation.

Altogether, both historical and genetic evidence support the fact that Sundarbans tigers were connected by gene flow with other mainland tigers from peninsular India until very recent. Hence, we finally conclude that isolation from mainland tiger population; subsequent gene flow and local adaptation have jointly shaped the genetic architecture of Sundarbans tiger in this marshy ecosystem.

### Conservation implications

This study gives the first description of the genetic diversity and population structure of Sundarbans tigers and as such should be used for their conservation management. A closer look at the population genetic pattern inferred from our mitochondrial and microsatellite data analyses suggests that Sundarbans tigers are the most divergent group of Bengal tigers. Hence, it should be managed as a separate conservation unit (CU). According to the definitions of Ryder [13], Waples [94] and Moritz [14], Sundarbans tiger population meets ESU criteria because it has genetic and adaptive distinctiveness and shows reciprocal monophyly. In addition, Sundarbans tiger population also meets the Crandal et al. [15] criteria for a MU due to lack of ecological exchangeability and gene flow. Hence, general criteria to delineate conservation units, i.e. adaptive evolutionary conservation (AEC) as suggested by Fraser Bernanchez [11], would help to guide proper management efforts. Under AEC, we recommend that Sundarbans tigers need overarching conservation to preserve its unique morphological adaptation and genetic uniqueness, which is very important for its future survival as this population is in early stage of allopatric speciation. Therefore, Sundarbans tigers should be managed as an ESU under the AEC framework.

## Supporting Information

S1 Fig Population structuring using TESS program. (a) Selection of best possible number of genetic clusters on the basis of DIC criterion for BYM and CAR, detecting 5–6 genetic populations. (b) Individual assignment probabilities of Bengal tiger to genetic clusters using the model-based program of TESS (run K = 5 and 6).
(DOCX)

S1 File Demographic analysis. Demographic analyses methodology.
(DOCX)

S1 Table Null allele frequency. Observed Null allele frequency.
(DOCX)
