# Correction: Tigers of Sundarbans in India: Is the Population a Separate Conservation Unit?

**DOI:** 10.1371/journal.pone.0131135

**Published:** 2015-06-24

**Authors:** The PLOS ONE Staff

## Notice of Republication

This article was republished on May 28, 2015, to replace Figs. 1–4, which were from a different article. The publisher apologizes for the error. Please download this article again to view the correct version.
