# Supplementary figures and images for: Tigers of Sundarbans in India: Is the Population a Separate Conservation Unit?
Source: PLoS One. 2015 Apr 28;10(4):e0118846. doi: 10.1371/journal.pone.0118846 (PMC4412631; doi:10.1371/journal.pone.0118846)

**S1 Fig.**

A)

B)


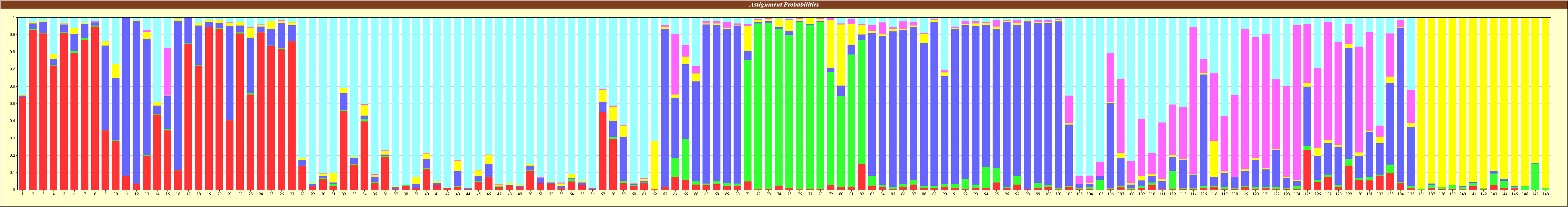

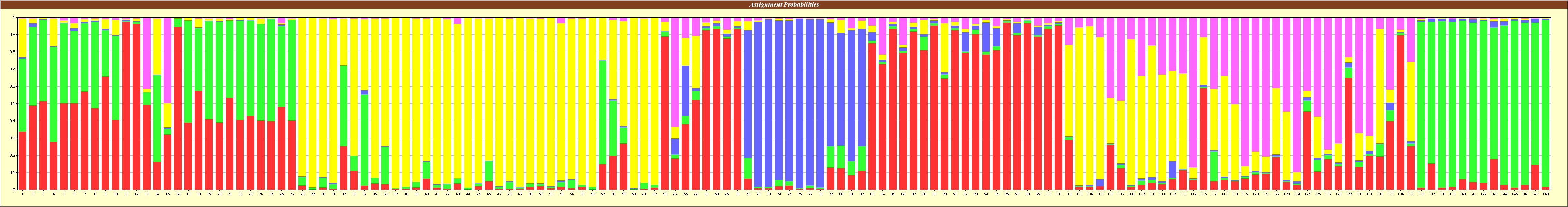


**Northern**

**Peninsular**

**Sundarbans**

K=6

K=5

Supplement: S1 Fig — (a) Selection of best possible number of genetic clusters on the basis of DIC criterion for BYM and CAR, detecting 5–6 genetic populations. (b) Individual assignment probabilities of Bengal tiger to genetic clusters using the model-based program of TESS (run K = 5 and 6). (DOCX) [file pone.0118846.s001.docx]
