# Supplementary material for: Tigers of Sundarbans in India: Is the Population a Separate Conservation Unit?
Source: PLoS One. 2015 Apr 28;10(4):e0118846. doi: 10.1371/journal.pone.0118846 (PMC4412631; doi:10.1371/journal.pone.0118846)
Supplement: S1 File — Demographic analyses methodology. (DOCX) [file pone.0118846.s002.docx]

**S1 File. Demographic analysis methodology.**

Bayesian skyline plots (Drummond *et al.* 2005) of mitochondrial effective population size through time were produced from the sequences of 1063 bp of ND2, ND5 and cytb genes of Sundarbans tigers, also including the sequences of northern and Peninsular populations by Mondol *et al.* 2009. We used the MCMC sampling algorithm implemented in program BEAST v. 1.8 (Drummond *et al.* 2012). The underlying population size function was fitted using the piecewise constant function. Substitution model obtained from MODELGENERATOR (HKY) was used to infer the ancestral trees. Markov chains were run for 20,000,000 generations and sampled every 2,000 generations with the first 2,000,000 generations discarded as burn-in. To scale the time and population size, the rate of molecular evolution was calibrated using the estimate (2.29 x 10^-8^ /year) from Luo *et al.* 2004.

We used the program DIYABC v. 2.0.4 (Cornuet *et al.* 2014) to explore the divergence times of the populations and effective population size using microsatellite data. DIYABC is a coalescence-based program that infers the population history by looking backwards in time to examine genealogy of alleles until reaching the most recent common ancestor, using approximate Bayesian computation algorithm. This algorithm simulates data sets for each of a specified set of scenarios of historic and /or demographic events, and compares summary statistics from these with the summary statistics of the observed data. The posterior probability and distribution of parameters of each scenario are estimated and alternative scenarios can be compared. Here, we used the information derived from eight microsatellite loci in 148 individuals, sampled from northern (Pop 1), Peninsular (Pop 2) and Sundarbans (Pop 3) tiger populations. We explored different demographic scenarios with splitting and admixture events to explain today´s observed tiger sub-populations.

After exploring several preliminary runs with different scenarios, we constructed three possible scenarios. Scenario 1 corresponds to a recent divergence of Sundarbans and northern tiger population from an ancestral large population. Scenario 2 corresponds to a recent divergence of all current tiger populations, including Sundarbans from an ancestral large population. In scenario 3, the Sundarbans tiger population originated from an admixture between individuals derived from ancestral Peninsular and northern tiger populations. The generalized stepwise model of mutation was applied with default values (GSM) (Fu and Chakraborty 1998 & Estoup *et al.* 2012). For all simulations, wide and large priors were used for all parameters (e.g. 10 - 100,000 for ancestral effective population size) and conditions set for the order of historic events. Similarly, the priors used for estimating the divergence time were 10 – 2000 (t1) and 10 – 5000 (t2) generations (a generation time of five years was used (Smith & McDougal 1991). For the three explored scenarios, 3,000,000 data sets were simulated.

The scenario most similar to the observed data was identiﬁed through the direct and logistic regression rejections steps of the algorithm and used for ABC estimation of parameters. We used all the available summary statistics for one sample: mean number of alleles, mean heterozygosity, mean size variance and mean Garza-Williamson’s *M,* and for two sample: mean number of alleles, mean heterozygosity and mean size variance, *F_ST_*, classification index, shared allele distance and (dμ)2 distance. For each simulated data set, 4 within population and 7 among population summary statistics were calculated. In addition, the confidence in scenario choice was evaluated by estimating type I and type II errors.

**Supplementary Method S3 References**

1. Drummond AJ, Rambaut A, Shapiro B, Pybus OG (2005) Bayesian Coalescent Inference of Past Population Dynamics from Molecular Sequences. Mol Biol Evol 22: 1185-1192.
2. Drummond AJ, Suchard MA, Xie D, Rambaut A (2012) Bayesian Phylogenetics with BEAUTI and the BEAST 1.7. Mol Biol Evol 29: 1969-1973.
3. Luo SJ, Kim JH, Johnson WE, Van der Walt J, Martenson J, Yunki N, Miquelle DG et al. (2004) Phylogeography and genetic ancestry of tigers (Panthera tigris). PLoS Biology 2: 2275–2293.
4. Mondol S, Karanth KU, Ramakrishnan U (2009) Why the Indian subcontinent holds the key to global tiger recovery. PLoS Genetics 5(8): e1000585.
5. Cornuet JM, Veyssier J, Pudlo P, Dehne-Garcia A, Gautier M, Leblois R, Marin JM Estoup A (2014) DIYABC v2.0: a software to make approximate Bayesian computation inferences about population history using Single Nucleotide Polymorphism, DNA sequence and microsatellite data. Bioinformatics 30: 1187-1189.
6. Fu YX, Chakraborty R (1998) Simultaneous estimation of all the parameters of a stepwise mutation model. Genetics 150:487-497.
7. Estoup A, Lombaert E, Marin JM, Guillemaud T, Pudlo P, Robert CP, Cornuet JM (2012). Estimation of demo-genetic model probabilities with Approximate Bayesian Computation using linear discriminant analysis on summary statistics. Molecular Ecology Resources 12(5):846–855.
8. Smith JLD, McDougal C (1991) The contribution of variance in lifetime reproduction to effective population size in tigers. Conservation Biology 5(4):484 – 490.
