# Supplementary material for: Tigers of Sundarbans in India: Is the Population a Separate Conservation Unit?
Source: PLoS One. 2015 Apr 28;10(4):e0118846. doi: 10.1371/journal.pone.0118846 (PMC4412631; doi:10.1371/journal.pone.0118846)
Supplement: S1 Table — Observed Null allele frequency. (DOCX) [file pone.0118846.s003.docx]

**S1 Table**

| Loci used | Central | Northern | STR | Overall (NI=CI=STR |
| --- | --- | --- | --- | --- |
| PttA2 | 0.0898 | 0.3158 | 0.2325 | 0.2252 |
| PttA4 | 0.2223 | 0.0539 | -0.0833 | 0.1315 |
| Pttc6 | 0.0531 | 0.1612 | -0.0564 | 0.0945 |
| FCA304 | 0.1541 | 0.0591 | -0.104 | 0.0916 |
| FcCA272 | 0.2297 | 0.0561 | 0.0545 | 0.1331 |
| PUN327 | 0.3876 | 0.2397 | 0.6308 | 0.3416 |
| PttE5 | 0.573 | 0.261 | 0.1065 | 0.4403 |
| F4 | 0.2926 | 0.1634 | 0.318 | 0.2327 |
| Pun82 | 0.3639 | 0.3765 | -0.0169 | 0.3723 |

Null allele frequency estimates by MICROCHECKER.

| Loci used | BTR | Kanha | Palamau | Panna | Pench | Rajaji | CTR | Dudhwa |
| --- | --- | --- | --- | --- | --- | --- | --- | --- |
| PttA2 | 0.5479 | -0.1197 | 0.3675 | 0.2376 | 0.2274 | -0.08 | 0.0778 | 0.3846 |
| PttA4 | -0.0117 | -0.062 | 0.1668 | 0.2119 | 0.061 | 0.2618 | 0.1909 | -0.0692 |
| Pttc6 | 0.3883 | 0.1181 | -0.1011 | 0.3319 | 0.2032 | -0.1433 | 0.1021 | 0.1974 |
| FCA304 | -0.1325 | 0.1121 | 0.0263 | 0.1274 | -0.0303 | 0.3319 | 0.0037 | 0.5123 |
| FcCA272 | -0.13 | 0.4111 | 0.0847 | -0.0692 | -0.0175 | 0.2557 | 0.1274 | 0.3104 |
| PUN327 | 0.0218 | 0.3136 | 0.2951 | 0.1735 | 0.1492 | 0.7125 | 0.0722 | 0.7622 |
| PttE5 | 0.1735 | 0.2277 | 0.03 | 0.0732 | 0.3721 | 0.7832 | 0.3295 | 0.5499 |
| F4 | -0.0634 | 0.1357 | 0.6823 | 0.1181 | -0.1025 | 0.4558 | 0.1513 | 0.6395 |
| Pun82 | 0.3636 | 0.2531 | 0.5891 | 0.1668 | 0.2002 | 0.4694 | 0.3051 | -0.1585 |
